# Retroperitoneal Mass, Abdominal Pain, and Bilateral Hydronephrosis Caused by Testicular Seminoma: A Case Report and Review of the Literature

**DOI:** 10.7759/cureus.81391

**Published:** 2025-03-28

**Authors:** Olaniyi Fadeyi, Saviz Saghari, Ali Esmaeili, Apoorva Cherukuri, Yunfei Wei

**Affiliations:** 1 Internal Medicine, West Anaheim Medical Center, Anaheim, USA; 2 Oncology, West Anaheim Medical Center, Anaheim, USA

**Keywords:** bilateral hydronephrosis, bilateral orchiectomy, cryptorchidism, testicular germ cell tumors, testicular seminoma

## Abstract

Testicular germ cell tumors are very common among young men. Most cases are diagnosed at early stages. However, in some instances, the patient may present with metastasis along with associated symptoms. Complaints after testicular cancer metastasis are diverse and vague. Consequently, it is important for clinicians to be familiar with various forms of presentation seen in testicular cancers. Here, we report a case of a 44-year-old patient who presented to the hospital with complaints of abdominal pain, testicular swelling, and bilateral lower extremity edema. This patient was subsequently diagnosed with testicular seminoma and severe bilateral hydronephrosis. Oncology and Urology were consulted for further management. In this report, we review this rare case and discuss different ways testicular seminoma cases could present clinically.

## Introduction

Testicular cancer is a common malignancy seen among male individuals aged 15-35 years [1]. Young men with certain risk factors, which may include undescended testis, family history of testicular cancers, previous history of testicular cancers, sexually transmitted diseases, and trauma, are prone to developing this malignancy. Incidence of testicular tumors is noticeable among non-Hispanic White men [2]. According to literature, the occurrence of testicular cancers in patients with a history of undescended testis is significantly higher than the general population [3]. Although testicular seminoma responds well to chemotherapy, late presentation may constitute a significant challenge to management. Previous studies have shown that timely diagnosis of primary testicular cancer before metastasis could bring about a 99% cure rate as opposed to a 74% cure rate for already metastasized testicular cancer at diagnosis [4]. Lack of symptoms at the early stages of disease, poor self-examination precision, and ignorance about the significance of testicular swelling may result in late presentation [5]. It is important to note that there are currently no generally acceptable guidelines to screen for testicular cancer. Testicular self-examination is only recommended by the United States Preventive Services Task Force (USPSTF) in select cases, as it has not been proven to improve outcomes [6]. Regardless of the common presentation of testicular cancers as testicular mass and swelling, several forms of atypical presentations have been described in the literature. Herein, we present a case of a 44-year-old male patient who was diagnosed with testicular seminoma and bilateral hydronephrosis. In addition, various atypical forms of presentation already documented in literature are highlighted.

## Case presentation

A 44-year-old male patient with no significant past medical history presented to the Emergency Department (ED) in May 2024 with complaints of abdominal pain, abdominal distention, and significant weight loss. The patient also reported bilateral lower extremity edema, challenges with ambulation, difficulty urinating, and persistent scrotal edema, which had been present for three years. He denied dysuria symptoms, hematuria, tobacco smoking, alcohol abuse, and recreational drug use. The patient had no known family history of testicular cancer, and there was no documented history of undescended testis. Physical examination was significant for scrotal edema, 3+ pitting edema in the lower extremities, and a severely distended abdomen without tenderness. The patient appeared cachectic. Vital signs were stable except for mild tachycardia. Laboratory results were largely unremarkable except for elevated creatinine, transaminitis, abnormal total protein levels, and elevated alkaline phosphatase (Table 1).

**Table 1:** Laboratory results with the respective reference ranges *Significant laboratory results WBC: white blood cell count; HCO_3_: bicarbonate; BUN: blood urea nitrogen; AST: aspartate aminotransferase; ALT: alanine aminotransferase; LDH: lactate dehydrogenase; bHCG: beta-human chorionic gonadotropin

Laboratory parameters	Results	Reference range
WBC	7.1	4.8-10.8 X 10^3^/uL
Hemoglobin	9.8	13.7-17.5 g/dL
Hematocrit	29	38.8%-50%
Platelets	290	150-450 X 10^3^ u/L
Sodium	132	136-145 mmol/L
Potassium	4.3	3.5-5.3 mmol/L
Chloride	97	96-106 mEq/L
HCO_3_	23.5	22-26 mmol/L
BUN	39	6-24 mg/dL
Creatinine	1.36	0.6-1.2 mg/dL
Blood glucose	81	70-140 mg/dL
AST*	92	8-48 U/L
ALT	8	7-55 U/L
Alkaline phosphatase*	417	30-147 IU/L
Total protein*	9.2	6.4-8.4 g/dL
Albumin*	2.4	3.6-5.1 g/dL
Lipase	130	0-160 U/L
LDH*	1986	40-280 U/L
bHCG*	617	<5 IU/L

**Figure 1:**
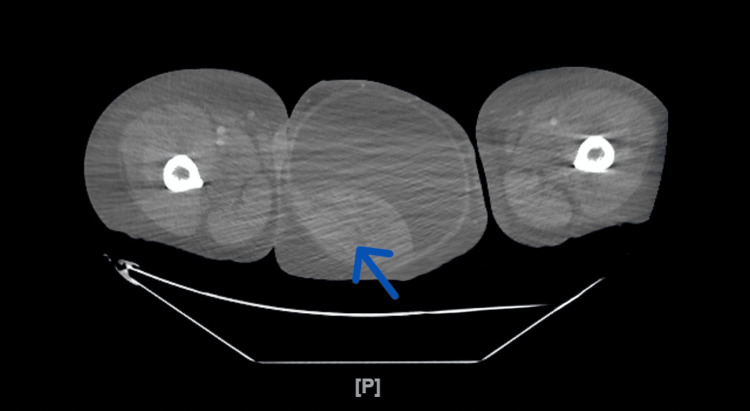
CT of the abdomen and pelvis with contrast showing left testicular heterogenous mass (blue arrow)

**Figure 2:**
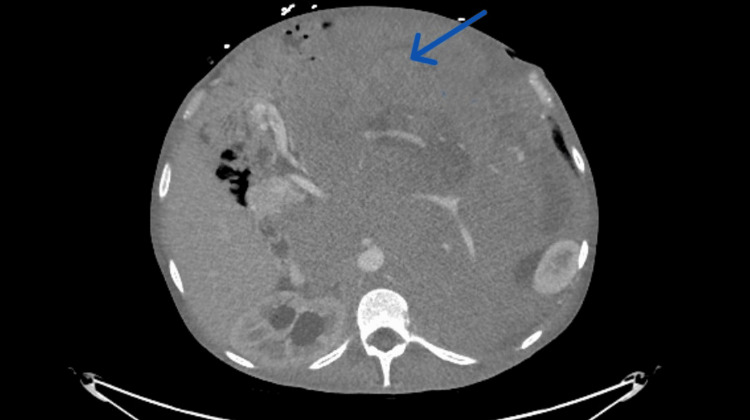
CT of the abdomen and pelvis with contrast showing very large retroperitoneal mass in the abdomen (blue arrow)

Due to abdominal distention and scrotal edema, a contrast-enhanced CT of the abdomen and pelvis was completed. Results revealed a left testicular heterogeneous mass (Figure 1) and a very large heterogeneous retroperitoneal mass occupying the abdomen and pelvis (Figure 2).

**Figure 3:**
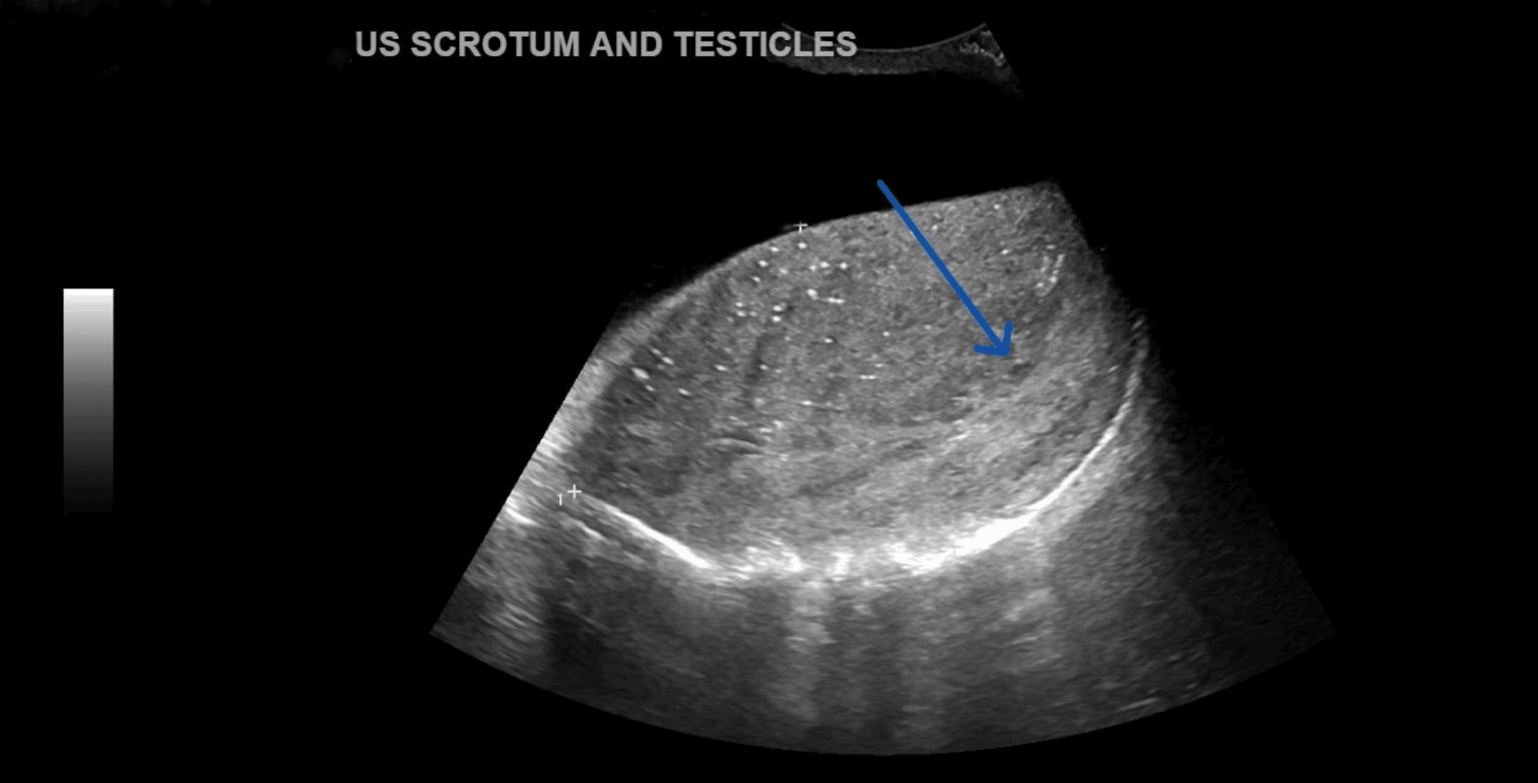
Testicular ultrasound showing left-sided testicular mass (blue arrow) US: ultrasound

Also, the left-sided testicular ultrasound result was significant for testicular mass as shown (Figure 3).

A contrast-enhanced chest CT did not reveal any evidence of intrathoracic metastasis. Lower extremity Doppler ultrasound was unremarkable for any deep vein thrombosis (DVT). Oncology and Urology services were consulted. Tumor markers showed elevated lactate dehydrogenase (LDH) and beta-human chorionic gonadotropin (bHCG), along with normal alpha-fetoprotein (AFP). A retroperitoneal mass core biopsy was completed, and histopathologic findings were consistent with testicular seminoma. Our patient was diagnosed with stage IIIB testicular seminoma, given the metastatic spread to the retroperitoneal space. Bilateral nephrostomy tubes were placed to relieve hydronephrosis. The patient improved clinically and was subsequently discharged from the hospital in June 2024. 

After discharge, the patient was promptly scheduled for left-sided radical inguinal orchiectomy by Urology. Chemotherapy was initiated after the procedure. Of note, the patient declined sperm cryopreservation. He underwent three cycles of bleomycin, etoposide, and cisplatin. Upon completion, the patient continues to follow up with Oncology for routine physical exams, blood tests, and imaging scans to detect recurrence and new metastasis.

## Discussion

Seminoma and non-seminoma are predominant forms of tumors of the male reproductive cells. Testicular seminoma has been described as a neoplasm that affects the germinal epithelium of the seminiferous tubules [7]. Despite the malignant potential, testicular seminoma has an excellent cure rate if detected early. However, late presentation and poor compliance with treatment may worsen this disease and result in poor outcomes [8]. Although testicular cancer could present as a painless testicular swelling, presentation may also be variable and dependent on the stage at diagnosis. For instance, our patient presented with abdominal distension, abdominal pain, testicular swelling, bilateral hydronephrosis, and lower extremity edema. Although common sites of metastasis for testicular germ cell tumors include the lungs, bone, liver, and lymph nodes, this patient had abdominal metastasis, which is quite uncommon. Previous studies have shown that abdominal metastasis is an unusual occurrence seen in less than 1% of patients with pure testicular seminoma [9,10]. Regardless of the effectiveness of chemotherapy in treating metastatic testicular seminoma, abdominal metastasis poses a considerable challenge to management and treatment [11].

Apart from abdominal distension and bilateral hydronephrosis seen in our patient, it is interesting to note that testicular seminoma could also present in other ways, as previously documented in the literature. For instance, Shogbesan et al. [12] documented a case of severe anemia in a 26-year-old patient who had perforation of the duodenum by retroperitoneal metastasis from stage IIIA testicular seminoma. This patient initially presented to the ED with severe anemia secondary to gastrointestinal bleeding. Esophago-gastro-duodenoscopy (EGD) was significant for duodenal mass. Also, CT of the abdomen and pelvis showed a soft tissue mass in the retroperitoneal space extending into the second and third portion of the duodenum. Further work-up revealed a prior history of testicular trauma, and the physical exam was significant for scrotal swelling. Elevated bHCG and LDH, along with normal AFP, were noted. Histopathology result of the duodenal mass was consistent with a malignant germ cell tumor. The patient underwent orchiectomy. The pathology result was consistent with stage IIIA seminoma.

Furthermore, Filiz et al. [13] reported a case of testicular seminoma presenting as acute abdomen due to ileal perforation. The patient was a 48-year-old man who presented to the ED with complaints of right lower quadrant abdominal pain and palpable abdominal mass. CT of the abdomen and pelvis revealed a large mass in the right lower quadrant. The chest X-ray was significant for free subdiaphragmatic air, which is pathognomonic for hollow viscus perforation. Prompt right hemicolectomy and ileal resection were completed. The subsequent pathology result was significant for seminoma secondary to undescended testis.

Again, retroperitoneal seminoma presenting as testicular pain was described by Joshi and Sivarajah [14]. The patient was a 46-year-old man who presented with right-sided testicular pain. Testicular torsion and epididymitis were ruled out. Meanwhile, results of the CT scan of the abdomen and pelvis with contrast showed a para-aortic mass displacing the duodenum and inferior vena cava (IVC), suggestive of a retroperitoneal tumor. CT-guided core biopsy of the para-aortic mass was done. Seminoma was diagnosed based on morphological appearance and immunohistochemical staining.

Similarly, González-Padilla et al. [15] documented a case of metastatic testicular cancer presenting as hematuria and renal colic. The patient was a 25-year-old man who came to the ED with complaints of right-sided flank pain. Renal ultrasound showed right-sided hydronephrosis without ureteral lithiasis. Further evaluation with CT abdomen and pelvis revealed a retroperitoneal mass infiltrating the right ureter. The testicular ultrasound result was significant for testicular mass. Right radical orchiectomy and placement of a double-J stent were completed. Elevated LDH and bHCG, along with normal AFP, were noted. The pathology result was significant for testicular cancer. Sperm cryopreservation was done before initiation of chemotherapy.

In another study, Raup et al. [16] described a case of testicular seminoma presenting as a renal mass and IVC thrombus in a 34-year-old male patient who came to the ED with complaint of back pain. Abdominal MRI was significant for an 8 cm renal mass with IVC thrombus and retroperitoneal lymphadenopathy. Testicular ultrasound revealed a hypoechoic lesion in the left testis. The renal mass biopsy result was consistent with metastatic melanoma. After orchiectomy, the patient was treated with systemic chemotherapy and placement of an IVC filter cephalad to the thrombus.

Also, Rosenblum et al. [17] documented an interesting case of a retroperitoneal tumor entrapping nerve ganglion and masquerading as a paraganglioma. The patient was a 57-year-old man who presented with worsening blood pressure, flushing, tachycardia, and a disturbing headache. CT of the abdomen and pelvis showed a right retroperitoneal mass with adjacent lymphadenopathy. Biochemical evaluation was equivocal, prompting a need for surgical evaluation. Paroxysmal symptoms were resolved after surgery. The histopathology result was significant for seminoma and an entrapped large ganglion within the tumor. This case suggested that mechanical pressure on a nerve ganglion from a retroperitoneal seminoma could be considered in a case indicative of paraganglioma, but without classic laboratory and imaging findings.

As highlighted above, atypical presentations of testicular seminoma can masquerade as other pathologies and thereby bring about missed or late diagnosis. Regardless of the presentation of testicular seminoma, radical inguinal orchiectomy and chemotherapy remain the standard of care. Histopathology examination of the specimen is very useful to confirm the diagnosis and identify the specific germ cell tumor [18]. CT of the chest, abdomen, and pelvis along with MRI of the brain are useful for staging. The management of our patient adhered to the standard of care both during hospitalization and after discharge. 

## Conclusions

Testicular seminoma can present in different ways, as described in previous studies documented in the literature. Elevated LDH and bHCG, along with normal AFP, are typical findings in testicular seminoma. Regardless of the form of presentation, clinicians should include testicular cancer as part of the differential diagnoses when evaluating young and middle-aged male patients with unexplained hydronephrosis and retroperitoneal masses. It is important to maintain a high index of suspicion when evaluating these patients, as testicular seminoma remains a curable disease, particularly when detected at early stages. Late diagnosis, alongside metastasis at presentation, may cause significant morbidity and mortality for patients. Radical inguinal orchiectomy and chemotherapy remain the standard of care.
